# Effects of Supplementing Intestinal Autochthonous Bacteria in Plant-Based Diets on Growth, Nutrient Digestibility, and Gut Health of Bullfrogs (*Lithobates catesbeianus*)

**DOI:** 10.3389/fmicb.2021.739572

**Published:** 2021-10-05

**Authors:** Zhe Wang, Chunxiao Zhang, Kangle Lu, Kai Song, Xueshan Li, Ling Wang, Samad Rahimnejad

**Affiliations:** ^1^Xiamen Key Laboratory for Feed Quality Testing and Safety Evaluation, Fisheries College, Jimei University, Xiamen, China; ^2^South Bohemian Research Center of Aquaculture and Biodiversity of Hydrocenoses, Faculty of Fisheries and Protection of Waters, University of South Bohemia in České Budějovice, České Budějovice, Czechia

**Keywords:** *Lithobates catesbeianus*, autochthonous bacteria, gut structure, gut microbiota, soybean meal-based diet

## Abstract

Poor utilization efficiency of plant protein diets always leads to intestinal barrier dysfunction and growth inhibition in animals. Probiotics have shown promise in improving growth performance and gut health of the host. However, obtaining the host-beneficial probiotic from thousands of bacterial phylotypes is challenging. Here, four intestinal autochthonous bacteria were isolated from fast-growing bullfrog after a 60-day feeding on a soybean meal (SM)-based diet. Another feeding trial was conducted to evaluate the effects of supplementing these strains in an SM-based diet on growth, nutrient digestibility, immunity, and gut health of bullfrog. A high-SM basal diet was used as a non-supplemented control group (NC), and four other diets were prepared by supplementing the basal diet with 1 × 10^7^ CFU/g of *Bacillus siamensis*, *Bacillus tequilensis* (BT), *Bacillus velezensis*, and *Lactococcus lactis* (LL). Results showed that weight gain, feed efficiency, nitrogen retention, and apparent digestibility coefficients of dry matter and protein were significantly higher in the LL group compared with the NC group (*p* < 0.05). Furthermore, compared with the NC group, both BT and LL groups showed markedly higher jejunal protease and amylase activities, serum complement 4 and immunoglobulin M levels, jejunal muscularis thickness (*p* < 0.05), and up-regulated expression of *il-10* and *zo-1* genes (*p* < 0.05). High-throughput sequencing revealed higher abundances of *Bacillus* and *Cetobacterium* in BT and LL groups, respectively, accompanied with decreased abundances of *Enterobacter* and *Escherichia–Shigella*. Besides, KEGG pathways related to metabolisms were significantly enhanced by the LL diet relative to the NC diet (*p* < 0.05). Overall, the beneficial effects of two frog-derived probiotics were determined: supplementation of *L. lactis* in SM-based diet promoted growth and nutrient digestibility; both *B. tequilensis* and *L. lactis* supplementation improved immune response and intestinal barrier function of bullfrogs.

## Introduction

Plant proteins are widely used as substitutes for expensive animal-derived protein sources in aquaculture and livestock sectors (Li Z.C. et al., 2017; Naylor et al., 2021). However, due to their general drawbacks such as low palatability, imbalanced amino acid profile, and presence of anti-nutritional factors (Liu et al., 2019; Peng et al., 2019; Yao et al., 2019), an excess proportion of plant proteins in diets usually induce immune dysfunction, gut inflammation, and subsequent growth inhibition in animals (Sahlmann et al., 2013; Wang et al., 2020).

Recently, probiotics have garnered significant attraction for disease prevention and growth promotion in aquaculture (Melo-Bolivar et al., 2021). Efficacy of probiotics is dictated by genetic, nutritional, and environmental factors, and the origin of the probiotic strains, accordingly some probiotics are only effective in specific animals (Sun et al., 2009). To avoid potentially harmful effects on the host and endogenous microbiota, intestinal autochthonous bacteria have more advantages over allochthonous bacteria in colonizing host’s intestinal mucosa and exerting physiological effects (Ringø et al., 2018). In addition, it has been reported that bacteria attached to the intestinal epithelial surfaces are more likely to be the real autochthonous bacteria (Denev et al., 2009). Bullfrog (*Lithobates catesbeianus*) has become one of the most economically valuable farmed amphibians worldwide. The evaluation of the cultivable indigenous microbiota from bullfrog specimens has already been performed as well as the study of the beneficial properties of some bacterial groups to advance in the design of probiotics to control both bacterial and fungal diseases (Montel Mendoza et al., 2012; Niederle et al., 2019). Some *in vivo* studies proposed the use of native or commercial lactic acid bacteria as potential probiotics for bullfrog hatcheries (de Clarla Dias et al., 2010; Pereira et al., 2016, 2018). However, information about the application of autochthonous probiotics isolated from intestinal mucosa of bullfrogs is scarce.

Growth rate of animals is determined by a series of factors such as ambient temperature, stocking density, available nutrients, etc. (Baltz et al., 1998; Nielsen, 2012). In addition, increasing studies showed that gut microbiota is deeply involved in various host physiological processes (Gao et al., 2018). Thus, it can be regarded as an “internal factor,” which confers multifaceted effects on the host. Remarkable growth difference usually occurs in farmed animals in breeding industry although they might be reared under the same environmental and dietary conditions. Sun et al. (2009) found that gut microbial composition differs in fast-growing (FG) and slow-growing (SG) groupers where higher abundances of potential pathogens were recorded in SG individuals. Similarly, Gui et al. (2017) reported that gut microbiota of FG chicken showed higher diversity and richness compared with the slow- and medium-growing chicken. Furthermore, a research on sea cucumber (*Apostichopus japonicus*) revealed that the differences in *Actinobacteria* abundance might be associated with the remarkable difference in body weight (Sha et al., 2016). These studies indicated that the growth of animals is closely linked to the gut microbiota. Although gut microbiota has long been researched through traditional and molecular techniques, information on intestinal microbiota–host crosstalk is scarce. Thus, recognition of the host-beneficial probiotics from thousands of bacterial phylotypes is challenging. Besides, as diet plays a key role in animals’ gut community, whether some sort of bacterial species enriched by a specific diet could in turn contribute to utilization of the same diet is uncertain.

Soybean meal (SM) has been widely used as a typical plant protein source in aquafeed. Previous studies conducted in our laboratory showed that high-SM diet caused intestine inflammation in bullfrog referred to as SM-induced enteritis (SBMIE), accompanied with poor growth and feed utilization (Ding et al., 2019; Yang et al., 2019). In the present study, initially a high-SM diet was fed to bullfrogs for 60 days. Presumably, the FG individuals might harbor a more favorable gut microbiota, which could adapt to the gut environment established by the SM-based diet, and this in turn contributes to the host’s growth. Then, the dominant gut bacterial species enriched in FG bullfrogs were isolated. A 58-day feeding trial was conducted to investigate the effects of supplementing these bacterial strains in a high-SM diet on growth performance, feed utilization, nutrient digestibility, immune function, and gut health of bullfrog.

## Materials and Methods

### Preparation of Candidate Bacterial Strains

A high-SM diet (Supplementary Table 1) was used to feed 108 bullfrogs of similar size (26.14 ± 0.21 g) for 60 days. After the feeding trial, bullfrogs were euthanized by destroying the spinal cord with a pin and weighed (Wang et al., 2020). Then, 12 heaviest bullfrogs and 12 lightest bullfrogs were sampled and grouped into FG and SG groups, respectively. The gut brush border membranes (mucosa) of bullfrogs from two groups were scraped with a coverslip on ice under sterile conditions, and then moved into sterile tubes. The mucosa samples were homogenized in normal saline solution (NSS, 0.7% NaCl). After gradient dilution, each dilution was evenly spread onto three different plates in triplicate, including nutrient agar (pH 7.3 ± 0.1), Man Rogosa Sharpe (pH 6.5 ± 0.2) agar, and Bacillus agar (pH 7.0 ± 0.2; Hopebio Technology Co. Ltd, China). The plates were incubated in normoxic incubator (33∘C for 24 h) for growth of aerobic and facultative anaerobic bacteria. Then, the bacterial colonies were divided into different types based on the colony characteristics of shape, structure, size, opacity, and color, and the colonies of each recognizable type was counted for determining number and occurrence rate [(the number of plates containing the strain)/(the number of all plates) × 100]. Then, three to five representatives of each colony type were streaked on corresponding plates repeatedly until pure cultures were obtained. Finally, a total of 35 representative isolates with different types were successfully isolated from agar plates, and the isolates with higher occurrence rates and numbers in the plates of the FG group compared with those of the SG group (Table 1) were picked up for gene sequencing. The bacterial DNA was extracted using bacterial genome DNA extraction kits (SBS Genetech, China) and sent for sequence analysis of the 16S rRNA gene by Majorbio Bio-pharm Technology Co., Ltd. (Shanghai, China). The 16S rRNA sequences were blast in Ezbiocloud to determine genetic homology.

**TABLE 1:** Species with a higher occurrence rate or number in plates of fast-growing (FG) group compared with those of slow-growing (SG) group.

**Species**	**FG**		**SG**	
	**Occurrence rate (%)**	**CFU/g**	**Occurrence rate (%)**	**CFU/g**
*Lactococcus lactis* subsp. *lactis*^a^	100.00	4.76 × 10^6^	50.00	5.93 × 10^5^
*Chryseobacterium pennipullorum* ^b^	75.00	3.93 × 10^6^	83.00	4.31 × 10^5^
*Bacillus velezensis* ^c^	66.67	2.12 × 10^6^	50.00	1.99 × 10^6^
*Bacillus siamensis* ^d^	50.00	4.75 × 10^4^	41.60	2.25 × 10^4^
*Escherichia fergusonii* ^e^	34.00	7.5 × 10^3^	–	–
*Bacillus tequilensis* ^f^	50.00	–^h^	–	–
*Microbacterium lacticum* ^g^	50.00	–	–	–

*Species were generally recorded in ^*a, g*^Man Rogosa Sharpe plates, ^*b, c, e*^Nutrient agar plates, and ^*d, f*^Bacillus agar plates; ^ h^invalid data.*

### Preparation of Experimental Diets

*Bacillus* species and a lactic acid bacterium were cultured in nutrient broth or MRS broth overnight. These cultures were then centrifuged at 4,000 × *g* for 5 min, and the bacterial pellets were suspended in sterile NSS after washing twice with NSS. The number of alive bacterial cells in the suspensions was determined by plate-counting method (Balestra and Misaghi, 1997). A basal diet (Supplementary Table 1) containing 55% SM was produced following the protocol described by Li et al. (2019). Four treated diets were prepared by supplementing the basal diet with 1 × 10^7^ CFU/g feed of bacterial suspension of *Bacillus siamensis* (BS diet), *Bacillus tequilensis* (BT diet), *Bacillus velezensis* (BV diet), and *Lactococcus lactis* (LL diet). The non-supplemented control diet (NC diet) was supplemented with equivalent sterile NSS, then all diets were encapsulated with a mixture of fish oil and soybean oil and dried in the shade. To ensure the viability of bacterial cells, diets were prepared every 2 weeks and stored at −20∘C until used.

### Feeding Trial

Bullfrogs were obtained from a commercial farm and transported to the fisheries laboratory of Jimei University (Xiamen, China). Before starting the experiment, bullfrogs were reared in an indoor aquarium (π × 160 × 80 cm) supplied with 4–6 cm of freshwater. All bullfrogs were fed the basal diet twice daily for 3 weeks to acclimate them to experimental conditions. Then, a total of 195 disease-free bullfrogs with homogenous size (initial mean body weight 29.61 ± 0.28 g) were equally divided into five groups with triplicates per group (13 bullfrogs per tank), and they were allocated into fifteen 10-L tanks. Bullfrogs within three tanks were randomly assigned to each dietary treatment and hand-fed to apparent satiation twice daily (8:00 and 18:00) for 58 days. After each feeding, the uneaten feeds were siphoned out and the water in each tank was entirely renewed with fresh water. Feces samples in each tank were collected as described by Lin et al. (2020) over the last 2 weeks for digestibility analysis. During the feeding period, a 12 h light/12 h dark photoperiod was maintained by fluorescent lamp, air temperature ranged from 28 to 32∘C, and water temperature ranged from 27 to 31∘C.

### Sample Collection

At the end of the feeding trial, bullfrogs were fasted for 24 h and euthanized by destroying the spinal cord with a pin. The number and total weight of bullfrogs in each tank were recorded for analyses of survival and final body weight. Three bullfrogs per tank were randomly sampled and frozen at −20∘C for body composition analysis. The abdomen of 10 additional bullfrogs in each tank was opened immediately with sterile scissors. Blood samples of the aforementioned bullfrogs were collected from ductus arteriosus using a sterile syringe, transported into sterile tubes, and kept at 4∘C overnight. Then, serum was separated after centrifugation (3,000 × *g*, 10 min, 4∘C) and stored at −80∘C for further analyses. Liver and hind legs were dissected from three bullfrogs per tank and weighed for calculations of hepatosomatic index (HSI) and hind leg index (HLI). The jejunum samples were collected from two bullfrogs per tank and fixed in Bouin’s solution for morphology analysis. Jejunum samples were collected from three bullfrogs per tank and kept at −80∘C for tissue RNA extraction and analysis of enzyme activity. Jejunum samples of two bullfrogs per tank were sampled and pooled for intestinal microbiota analysis.

### Chemical Composition

The crude protein, crude lipid, moisture, and ash contents in diet, whole body, muscle, and feces samples were analyzed according to the standard method of AOAC (2002). Moisture content was estimated by drying in an oven at 105∘C until a constant weight was reached. Crude protein (N × 6.25) was determined by the Dumas method (Gerhardt, Germany). Crude lipid content was quantified by ether extraction, and ash content was measured by the combustion method in a muffle furnace at 550∘C for 8 h. The mineral element content of samples was determined by inductively coupled plasma atomic emission spectroscopy (ICP-OES; Leeman, United States).

### Digestive Enzymes

Intestine samples were homogenized in 10 volumes (w/v) of NSS and centrifuged at 3,000 × *g* for 10 min. Then, the homogenized solution was collected and stored at 4∘C. The protease activity was measured by Folin-phenol method (Lowry et al., 1951). One unit of protease activity was defined as the amount of the hydrolysis of casein that liberated 1 μg of tyrosine per minute. Lipase and amylase activities were quantified using commercial kits (Nanjing Jiancheng Biological Company, China). One unit of lipase activity was defined as the amount of enzyme that hydrolyzes 1 μmol substrate per minute at 37°C. One unit of amylase activity was defined as the amount of protein that hydrolyzed 10 mg starch per 30 min at 37°C.

### Gut Barrier Function and Immune Parameters

Serum D-lactate concentration, complement 3 (C3), complement 4 (C4), and IgM levels were measured by competition method according to Syedbasha et al. (2016) using amphibian ELISA kits from Nanjing Jiancheng Biological Company (Nanjing, China). Serum diamine oxidase (DAO) and lysozyme (LZM) activities were determined by colorimetric method using commercial assay kits (Nanjing Jiancheng Bioengineering Institute, China). One unit of LZM activity was defined as the amount of enzyme needed to decrease absorbance at a rate of 0.001 min^–1^⋅ml^–1^ at 37∘C, and one unit of DAO activity was defined as 1 mmol ammonia formed per minute per milliliter of serum at 37∘C.

### Quantitative Real-Time PCR

Total RNA was extracted from jejunum of bullfrogs using FastPure Tissue Total RNA Isolation Kit (Vazyme Biotech Co., Ltd, China). The purity of the total RNA was analyzed in 1% agarose gel electrophoresis, and the concentration of the total RNA was quantitated using NanoDrop One Ultramicro spectrophotometer (Thermo Fisher, United States). Total RNA was reversely transcribed to cDNA by TransScript ALL-in-one First-Strand cDNA Synthesis Kit (TransGen Biotech Co., Ltd., China). Quantitative PCR was performed on ABI StepOne Plus (Thermal Cycler, United States). The primers of the gene (Supplementary Table 2) and PCR amplification program were designed as our previous research on bullfrogs (Ding et al., 2019; Lin et al., 2021). The relative expression levels of genes were calculated using 2^–ΔΔCt^ method.

### Gut Histology

Jejunal samples were stained with H&E following the standard histological procedures conducted by Servicebio Biotechnology Co., Ltd. (Wuhan, China). The micrographs were observed with a light microscope (Leica DM5500B, Germany), and Image J software was used for morphometric analysis.

### Illumina High-Throughput Sequencing

Bacterial DNA was extracted from bullfrog jejunum using HiPure Soil DNA Kits (Magen, China). The DNA yield was detected using Nanodrop 2000 (Thermo Scientific, United States). The amplicons of 16S rRNA gene V3–V4 region were extracted from 2% agarose gel, purified by the AxyPrep DNA Gel Extraction Kit (Axygen Biosciences, United States), and quantified using ABI StepOnePlus Real-Time PCR System (Life Technologies, United States). Then, the purified amplicons were paired-end sequenced on Illumina MiSeq PE 250 (Gene *Denovo* Biotechnology Co., Ltd., China).

Noisy sequences of raw tags were filtered by QIIME software to obtain high-quality clean tags. Clean tags were searched against the reference database to perform reference-based chimera checking using UCHIME algorithm. All chimeric tags were removed and finally obtained effective tags were used for further analysis. The effective tags were clustered into operational taxonomic units (OTUs) of ≥97% similarity using UPARSE software. The tag sequence with the highest abundance was selected as a representative sequence within each cluster. Venn analysis and principal coordinates analysis (PCoA) based on Jaccard distance matrix were performed in R project package. The representative sequences were classified into organisms using RDP classifier based on SILVA database with the confidence threshold value of 80%. The abundance statistics of each taxonomy were visualized using Krona. The KEGG pathway analysis of the OTUs was inferred using PICRUSt2 (Douglas et al., 2020). Analysis of function difference between groups was calculated by Welch’s *t*-test in R project package. The raw reads were uploaded to NCBI Sequence Read Archive database (accession number PRJNA747862).

### Calculation and Statistical Analysis

Weight gain (WG, %) = (W_1_ − W_0_)/W_0_ × 100
Feeding rate (FR, %/day) = W_*D*__/_(W_1/_2 + W_0_/2)/*t* × 100
Feed efficiency (FE) = (W_1_ − W_0_)/W_*D*_
Nitrogen retention ratio (NRR, %) = (W_1_ × P_1_ − W_0_ × P_0_)/(W_*D*_ × P_*D*_) × 100
Survival (%) = N_1_/N_0_ × 100
Hepatosomatic index (%) = liver weight/W_1_ × 100
Hind leg index (%) = hind leg weight/W_1_ × 100
Apparent digestibility coefficients (ADCs, %) = (1 − F_0/_D_0_ × (D_*Y*_/F_*Y*_)) × 100

Where W_1_ and W_0_ are the mean final and initial wet body weights, respectively; W_*D*_ is dry feed intake; *t* is feeding days; P_*D*_, P_0_, and P_1_ are the concentrations of crude protein in the diet, initial body, and final body, respectively; N_0_ and N_1_ are the initial and final number of bullfrogs, respectively; D_*Y*_ and F_*Y*_ are the concentration of yttrium in diet and feces, respectively; and D_0_ and F_0_ are the quantities of compositions in diets and feces, respectively.

The homogeneity of variances of data was tested before further analysis. The significance of difference between the FG group and the SG group was analyzed by Student’s *t*-test using SPSS 22.0. One-way ANOVA followed by Tukey multiple comparison test was used to identify differences among NC, BT, BV, BS, and LL groups. The values are presented as mean ± SEM and the differences were considered significant at *p* < 0.05.

## Results

### Preparation of Candidate Bacterial Strains

After the 60-day feeding on the SM-based diet, the FG group showed significantly higher WG than the SG group (*p* < 0.05; Supplementary Figure 1). Some species enriched in the gut of the FG group, but scarce in the gut of the SG group, and the species with higher occurrence rate and numbers in the FG group compared with the SG group are listed in Table 1. Three *Bacillus* species (*B. siamensis*, *B. tequilensis*, and *B. velezensis*) and a lactic acid bacterium (*L. lactis* subsp. *lactis*) were then selected for the subsequent feeding trial.

### Growth Performance, Feed Utilization, and Organosomatic Indices

Compared with the NC group, WG was significantly increased in bullfrogs fed the LL diet (*p* < 0.05), and intermediary WG values were observed in BS and BT groups (*p* > 0.05; Table 2). No marked difference was found in FR between the NC group and each treated group (*p* > 0.05). LL supplementation led to a remarkable increment of FE compared with the other groups (*p* < 0.05). Moreover, the highest NRR was found in the LL group, which was significantly higher than that in NC and BV groups (*p* < 0.05). No significant difference was observed in survival, HSI, and HLI among all groups (*p* > 0.05).

**TABLE 2:** Growth performance, feed utilization, organosomatic indices, and survival of bullfrog.

	**NC**	**BS**	**BT**	**BV**	**LL**
FBW (g)	160.27 ± 2.01^a^	165.82 ± 4.69^ab^	172.33 ± 3.68^ab^	160.64 ± 1.87^a^	180.09 ± 2.06^b^
WG (%)	443.87 ± 9.54^a^	463.08 ± 15.90^ab^	487.88 ± 12.07^ab^	440.26 ± 6.21^a^	503.55 ± 6.14^b^
FR (%/day)	2.44 ± 0.01^ab^	2.46 ± 0.02^b^	2.45 ± 0.00^b^	2.45 ± 0.01^b^	2.40 ± 0.01^a^
FE	0.97 ± 0.00^a^	0.98 ± 0.00^ab^	1.00 ± 0.01^b^	0.97 ± 0.00^a^	1.03 ± 0.00^c^
NRR (%)	33.81 ± 1.12^a^	35.93 ± 0.30^ab^	35.97 ± 1.13^ab^	31.75 ± 1.24^a^	39.55 ± 0.25^b^
Survival (%)	100.00 ± 0.00^a^	100.00 ± 0.00^a^	100.00 ± 0.00^a^	100.00 ± 0.00^a^	100.00 ± 0.00^a^
HSI (%)	4.73 ± 0.54^a^	5.52 ± 0.04^a^	5.36 ± 0.28^a^	5.33 ± 0.57^a^	5.09 ± 0.74^a^
HLI (%)	33.90 ± 3.97^a^	38.66 ± 0.50^a^	38.13 ± 0.09^a^	38.05 ± 0.51^a^	40.65 ± 0.41^a^

*^*a–c*^Mean values in the same row with different superscripts differ significantly (*p* < 0.05).*

*NC, control diet; BS, control diet containing 1 × 10^7^ CFU/g of *Bacillus siamensis*; BT, control diet containing 1 × 10^7^ CFU/g of *Bacillus tequilensis*; BV, control diet containing 1 × 10^7^ CFU/g of *Bacillus velezensis*; LL, control diet containing 1 × 10^7^ CFU/g of *Lactococcus lactis*; FBW, final body weight; WG, weight gain; FR, feeding rate; FCR, feed efficiency; NRR, nitrogen retention ratio; HSI, hepatosomatic index; and HLI, hind leg index.*

### Body Composition

Bullfrogs fed the LL diet exhibited markedly higher whole-body protein content than both NC and BV groups (*p* < 0.05; Table 3). Whole-body moisture content significantly decreased in the LL group compared with that in the BV group (*p* < 0.05). Higher protein but lower moisture contents were found in the muscle of the BT group compared with that of the BV group (*p* < 0.05). No significant changes were found in whole-body lipid and ash, and muscle lipid contents (*p* > 0.05).

**TABLE 3:** Body composition analysis (%, wet weight) of bullfrog.

	**NC**	**BS**	**BT**	**BV**	**LL**
**Whole body**					
Moisture	75.63 ± 0.65^ab^	74.73 ± 0.29^ab^	73.85 ± 0.68^ab^	75.99 ± 0.53^b^	73.24 ± 0.38^a^
Protein	14.31 ± 0.34^a^	15.12 ± 0.10^ab^	14.94 ± 0.48^ab^	13.71 ± 0.40^a^	15.95 ± 0.12^b^
Lipid	6.14 ± 0.14^a^	6.24 ± 0.08^a^	6.08 ± 0.12^a^	6.01 ± 0.14^a^	6.30 ± 0.16^a^
Ash	2.65 ± 0.08^a^	2.65 ± 0.06^a^	2.71 ± 0.07^a^	2.73 ± 0.12^a^	2.67 ± 0.04^a^
**Muscle**					
Moisture	77.75 ± 0.62^ab^	78.16 ± 0.42^ab^	76.37 ± 0.30^a^	78.54 ± 0.38^b^	76.77 ± 0.19^ab^
Protein	20.36 ± 0.57^ab^	19.92 ± 0.38^ab^	21.34 ± 0.28^b^	19.39 ± 0.37^a^	20.49 ± 0.23^ab^
Lipid	0.55 ± 0.02^a^	0.53 ± 0.02^a^	0.56 ± 0.00^a^	0.52 ± 0.01^a^	0.56 ± 0.01^a^

*^*a, b*^Mean values in the same row with different superscripts differ significantly (*p* < 0.05).*

*NC, control diet; BS, control diet containing 1 × 10^7^ CFU/g of *Bacillus siamensis*; BT, control diet containing 1 × 10^7^ CFU/g of *Bacillus tequilensis*; BV, control diet containing 1 × 10^7^ CFU/g of *Bacillus velezensis*; and LL, control diet containing 1 × 10^7^ CFU/g of *Lactococcus lactis*.*

### Nutrient Digestibility and Digestive Enzyme Activity

**FIGURE 1:**
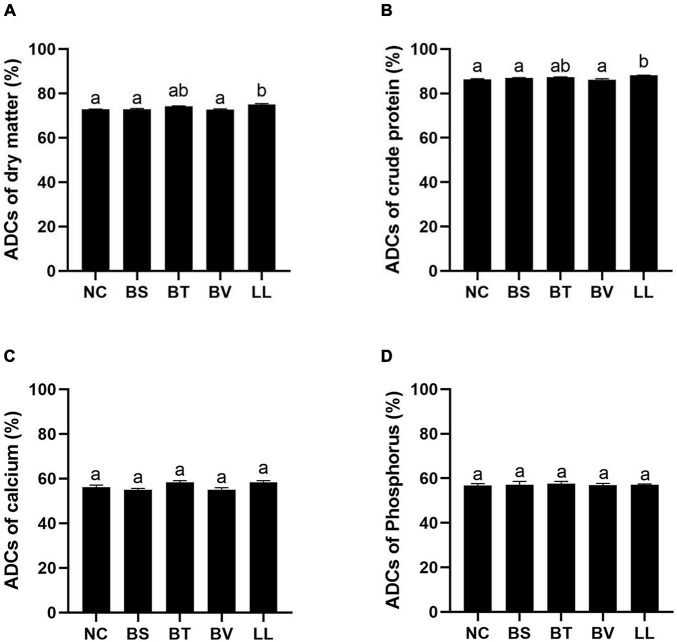
Apparent digestibility coefficients (ADCs) of nutrients: **(A)** dry matter, **(B)** protein, **(C)** calcium, and **(D)** phosphorus. Bars with different letters were significantly different (*p* < 0.05). NC, control diet; BS, control diet containing 1 × 10^7^ CFU/g of *Bacillus siamensis*; BT, control diet containing 1 × 10^7^ CFU/g of *Bacillus tequilensis*; BV, control diet containing 1 × 10^7^ CFU/g of *Bacillus velezensis*; and LL, control diet containing 1 × 10^7^ CFU/g of *Lactococcus lactis*.

**FIGURE 2:**
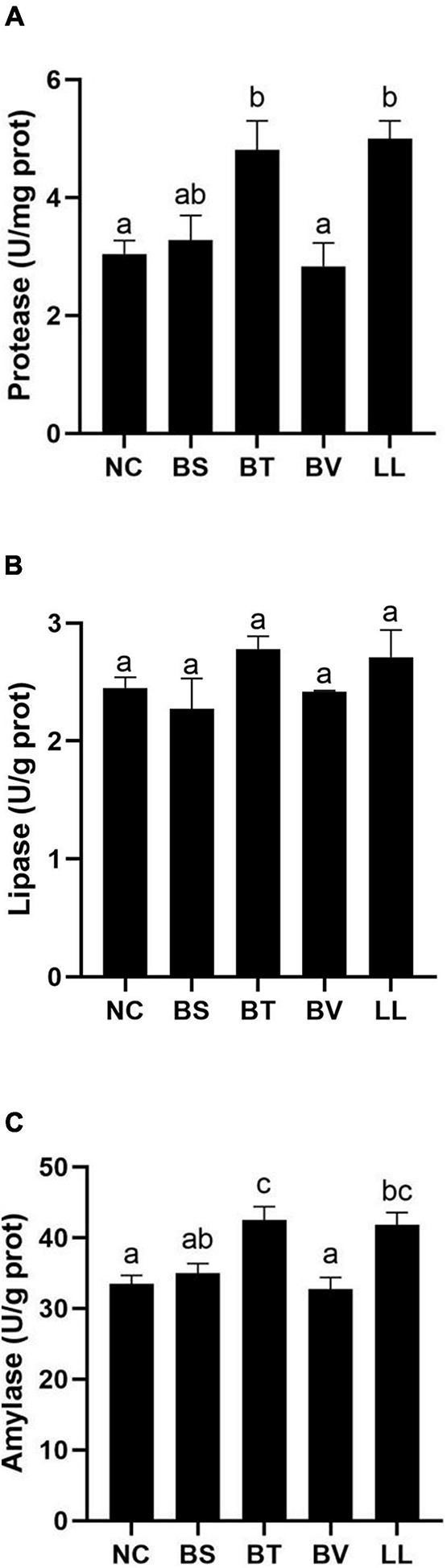
Digestive enzymes activity in jejunum of bullfrog: **(A)** protease, **(B)** lipase, and **(C)** amylase. Bars with different letters were significantly different (*p* < 0.05). NC, control diet; BS, control diet containing 1 × 10^7^ CFU/g of *Bacillus siamensis*; BT, control diet containing 1 × 10^7^ CFU/g of *Bacillus tequilensis*; BV, control diet containing 1 × 10^7^ CFU/g of *Bacillus velezensis*; and LL, control diet containing 1 × 10^7^ CFU/g of *Lactococcus lactis*.

Apparent digestibility coefficients of dry matter and crude protein were significantly increased in the LL group compared with those in NC, BS, and BV groups (*p* < 0.05; Figures 1A,B). There were no marked changes in ADCs of calcium and phosphorus among all groups (*p* > 0.05; Figures 1C,D). In addition, LL and BT groups exhibited profoundly higher jejunal protease and amylase activities than NC and BV groups (*p* < 0.05; Figures 2A,C). Bacteria supplementation did not significantly affect jejunal lipase activity (*p* > 0.05; Figure 2B).

### Humoral Immunity

Bullfrogs fed LL diet showed significantly higher serum LZM activity and C3 level compared with the NC group (*p* < 0.05; Table 4). Moreover, serum C4 and IgM levels were remarkably increased in both BT and LL groups relative to NC and BV groups (*p* < 0.05). BS and BV diets did not affect the aforementioned immune parameters in bullfrogs (*p* > 0.05).

**TABLE 4:** Humoral immune parameters including LZM, IgM, C3, and C4 of bullfrog.

	**NC**	**BS**	**BT**	**BV**	**LL**
LZM (U/ml)	632.96 ± 52.83^a^	651.68 ± 76.48^a^	812.73 ± 52.83^ab^	573.03 ± 45.41^a^	996.25 ± 103.86^b^
IgM (μg/ml)	359.88 ± 25.12^a^	394.18 ± 10.40^ab^	466.22 ± 10.49^b^	316.71 ± 10.76^a^	547.56 ± 22.33^c^
C3 (μg/ml)	141.26 ± 10.06^a^	155.4 ± 10.05^ab^	178.26 ± 12.09^ab^	138.27 ± 12.56^a^	202.28 ± 6.29^b^
C4 (μg/ml)	107.23 ± 4.04^a^	113.80 ± 5.42^a^	135.80 ± 3.86^b^	100.40 ± 6.21^a^	156.18 ± 2.60^b^

*^a–c^Mean values in the same row with different superscripts differ significantly (*p* < 0.05).*

*NC, control diet; BS, control diet containing 1 × 10^7^ CFU/g of *Bacillus siamensis*; BT, control diet containing 1 × 10^7^ CFU/g of *Bacillus tequilensis*; BV, control diet containing 1 × 10^7^ CFU/g of *Bacillus velezensis*; LL, control diet containing 1 × 10^7^ CFU/g of *Lactococcus lactis*; LZM, lysozyme; IgM, immunoglobulin M; C3, complement 3; and C4, complement 4.*

### Jejunal Morphology, Tight Junction Proteins, and Epithelial Permeability

**FIGURE 3:**
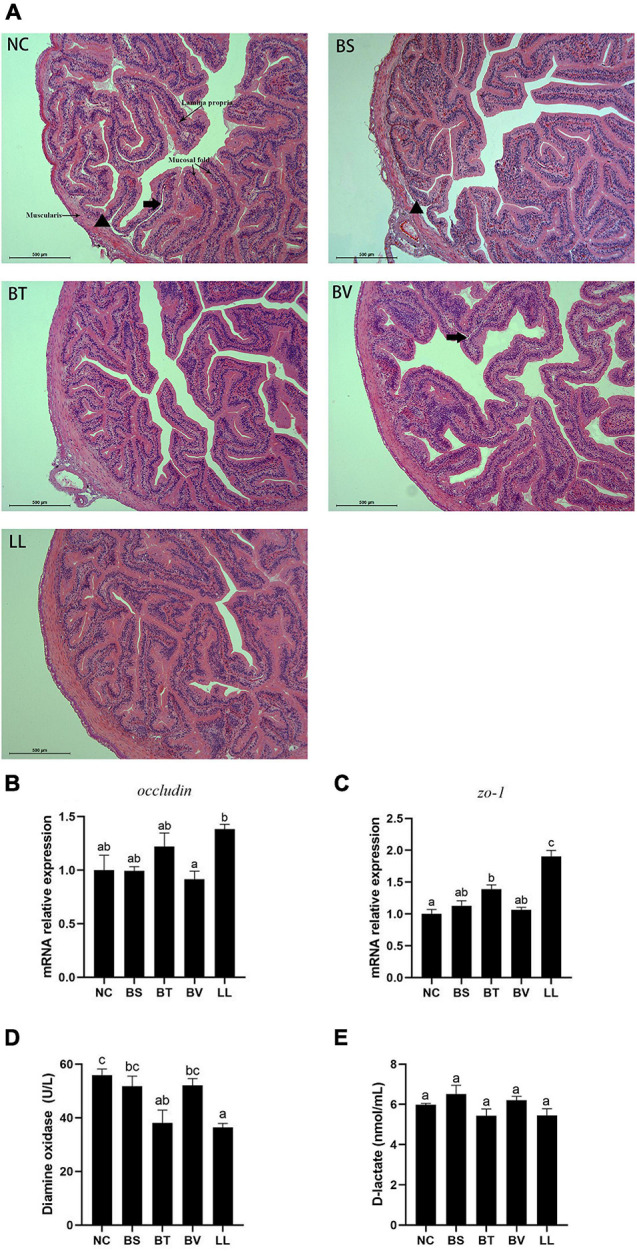
**(A)** Jejunal morphology of bullfrog using H&E staining. The arrow pointed to the separation between lamina propria and mucosa. The triangle pointed to the separation between submucosa and muscularis. **(B,C)** Expression of tight junction proteins genes (*occludin* and *zo-1*) in jejunum of bullfrog. **(D,E)** Intestinal epithelial permeability indicators (diamine oxidase and D-lactate) in serum of bullfrog. Bars with different letters were significantly different (*p* < 0.05). *zo*, zonula occludens; NC, control diet; BS, control diet containing 1 × 10^7^ CFU/g of *Bacillus siamensis*; BT, control diet containing 1 × 10^7^ CFU/g of *Bacillus tequilensis*; BV, control diet containing 1 × 10^7^ CFU/g of *Bacillus velezensis*; and LL, control diet containing 1 × 10^7^ CFU/g of *Lactococcus lactis*.

A typical SBMIE phenomenon was detected in the jejunal epithelium of NC, BS, and BV groups, including shrunken mucosal folds, narrow muscularis, and partial separation of tissue (Figure 3A). Conversely, the mucosal folds in the LL group were longer and curlier, accompanied by more branches. Besides, no obvious separation of tissue or degenerative changes were found in the BT and LL groups. Morphometric analysis of jejunum showed increased number (*p* < 0.05) and height (*p* > 0.05) of mucosal folds in the LL group compared with the NC group (Table 5). Furthermore, muscularis thickness in BT and LL groups was significantly higher than that in the other groups (*p* < 0.05). There was no significant difference in mucosal fold width and lamina propria thickness among groups (*p* > 0.05). In addition, the mRNA expression of tight junction protein *zo-1* (*p* < 0.05) and *occludin* (*p* > 0.05) increased in BT and LL groups compared with the NC group (Figures 3B,C). Moreover, compared with the NC group, BT and LL diets resulted in a significant reduction of serum DAO activity (*p* < 0.05; Figure 3D). However, no significant difference was found in serum D-lactate concentration among all groups (*p* > 0.05; Figure 3E).

**TABLE 5:** Jejunum morphological indices of bullfrog.

	**NC**	**BS**	**BT**	**BV**	**LL**
Number of mucosal folds	28.83 ± 1.45^ab^	30.0 ± 1.21^ab^	33.33 ± 1.86^bc^	25.67 ± 0.84^a^	36.17 ± 1.01^c^
Mucosal fold height (μm)	526.00 ± 21.34^ab^	517.83 ± 20.53^a^	572.00 ± 26.20^ab^	507.67 ± 21.62^a^	619.00 ± 22.39^b^
Mucosal fold width (μm)	157.00 ± 8.25^a^	168.83 ± 4.89^a^	166.33 ± 7.06^a^	148.67 ± 4.13^a^	169.17 ± 9.18^a^
Muscularis thickness (μm)	34.83 ± 3.46^a^	38.67 ± 2.23^a^	58.17 ± 2.36^b^	38.00 ± 4.86^a^	84.83 ± 7.42^c^
Lamina propria thickness (μm)	21.95 ± 1.76^a^	21.35 ± 1.60^a^	20.31 ± 0.96^a^	22.13 ± 2.53^a^	20.88 ± 1.88^a^

*^a–c^Mean values in the same row with different superscripts differ significantly (*p* < 0.05).*

*NC, control diet; BS, control diet containing 1 × 10^7^ CFU/g of *Bacillus siamensis*; BT, control diet containing 1 × 10^7^ CFU/g of *Bacillus tequilensis*; BV, control diet containing 1 × 10^7^ CFU/g of *Bacillus velezensis*; and LL, control diet containing 1 × 10^7^ CFU/g of *Lactococcus lactis*.*

### Jejunal Inflammatory Cytokines

**FIGURE 4:**
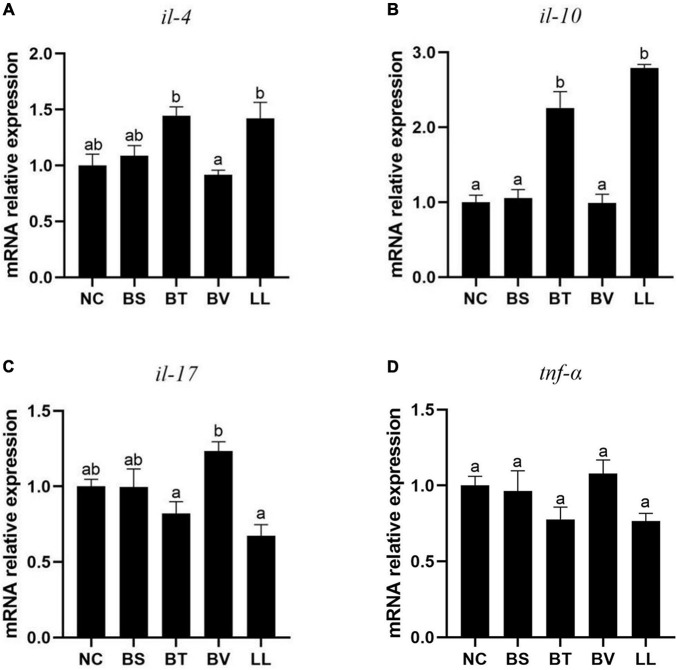
Expression of jejunal inflammatory cytokines of bullfrog, including **(A)** *il-4*, **(B)** *il-10*, **(C)** *il-17*, and **(D)** *tnf-*α. Bars with different letters were significantly different (*p* < 0.05). *il*, interleukin; *tnf*, tumor necrosis factor; NC, control diet; BS, control diet containing 1 × 10^7^ CFU/g of *Bacillus siamensis*; BT, control diet containing 1 × 10^7^ CFU/g of *Bacillus tequilensis*; BV, control diet containing 1 × 10^7^ CFU/g of *Bacillus velezensis*; and LL, control diet containing 1 × 10^7^ CFU/g of *Lactococcus lactis*.

Compared with the NC group, BT and LL supplementation led to up-regulated expression of anti-inflammatory cytokines including *il-10* (*p* < 0.05) and *il-4* (*p* > 0.05) genes (Figures 4A,B). No significant difference was found in the expression of pro-inflammatory cytokines (*il-17* and *tnf-*α genes) between the NC group and each of the treated groups (*p* > 0.05; Figures 4C,D).

### Jejunum Microbial Communities

**FIGURE 5:**
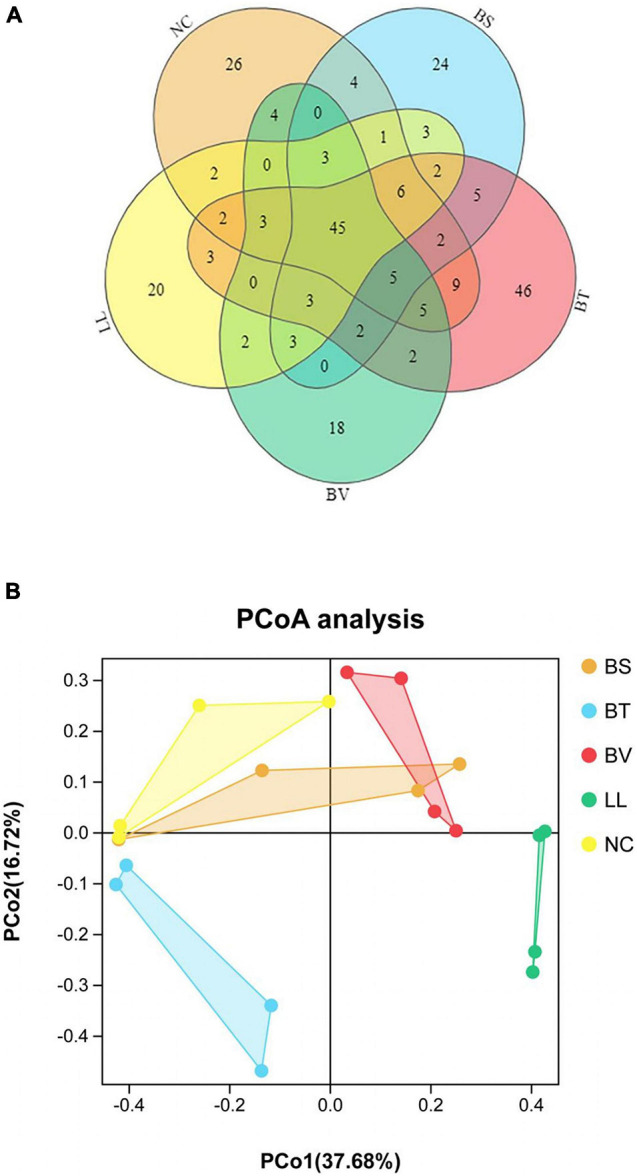
**(A)** Venn diagram and **(B)** principal component analysis (PCoA) based on Jaccard distance matrix of jejunal microbiota. NC, control diet; BS, control diet containing 1 × 10^7^ CFU/g of *Bacillus siamensis*; BT, control diet containing 1 × 10^7^ CFU/g of *Bacillus tequilensis*; BV, control diet containing 1 × 10^7^ CFU/g of *Bacillus velezensis*; and LL, control diet containing 1 × 10^7^ CFU/g of *Lactococcus lactis*.

A total of 2,160,089 effective tags were obtained from five groups, with an average of 108,004 ± 1,024 per sample. The resulting sequences were clustered into OTUs at 97% sequence identity. Thus, a total of 2,457 OTUs were found in five groups, with an average of 123 ± 5 per sample. Venn diagram of bacterial communities showed that the core OTUs of all groups was 45, and total OTUs of NC, BS, BT, BV, and LL groups were 199, 108, 140, 95, and 98, respectively, (Figure 5A). PCoA plot based on Jaccard distance matrix was executed to indicate the similarity of intestinal microbial communities among experimental groups. Some data points of NC, BS, and BV groups had overlap regions (Figure 5B), which were distinct from those of BT and LL groups.

**FIGURE 6:**
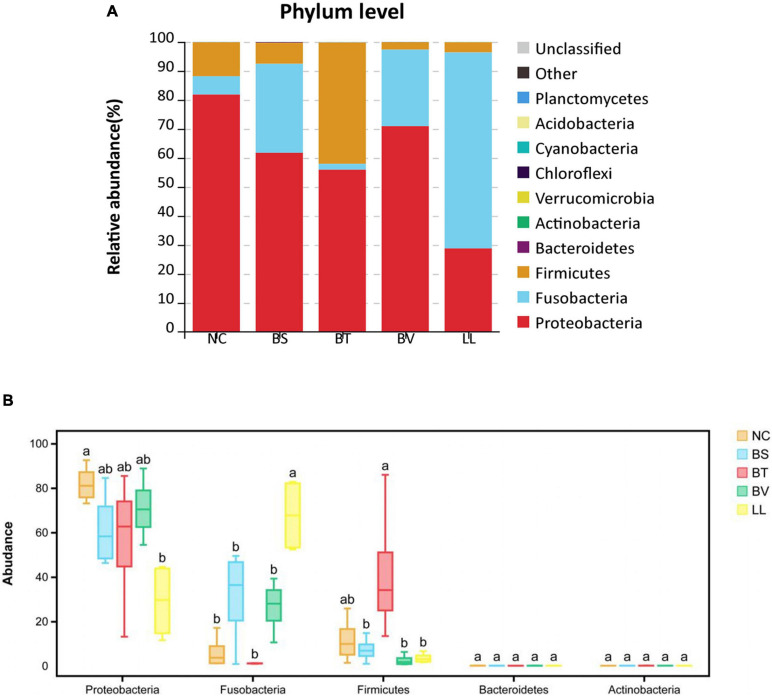
The relative abundance of major bacterial phyla in **(A)** barplot and **(B)** boxplot. Bars with different letters were significantly different (*p* < 0.05). NC, control diet; BS, control diet containing 1 × 10^7^ CFU/g of *Bacillus siamensis*; BT, control diet containing 1 × 10^7^ CFU/g of *Bacillus tequilensis*; BV, control diet containing 1 × 10^7^ CFU/g of *Bacillus velezensis*; and LL, control diet containing 1 × 10^7^ CFU/g of *Lactococcus lactis*.

**FIGURE 7:**
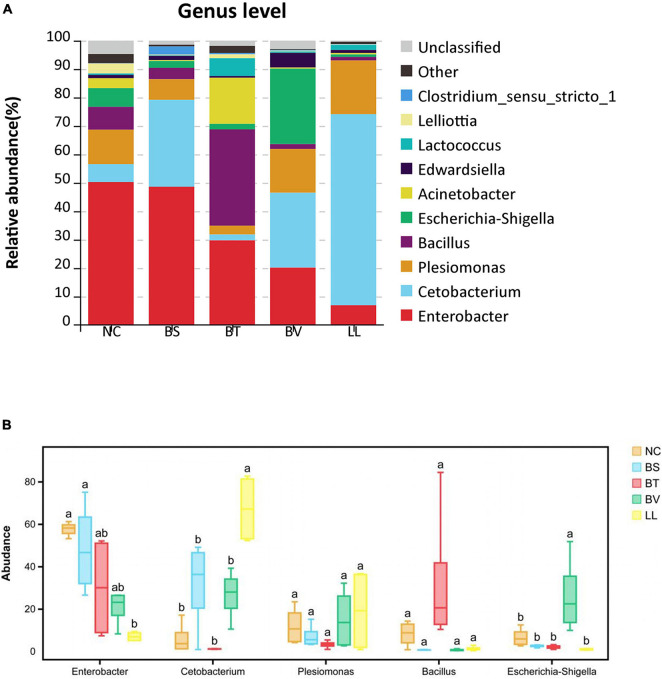
The relative abundance of major bacterial genera in **(A)** barplot and **(B)** boxplot. Bars with different letters were significantly different (*p* < 0.05). NC, control diet; BS, control diet containing 1 × 10^7^ CFU/g of *Bacillus siamensis*; BT, control diet containing 1 × 10^7^ CFU/g of *Bacillus tequilensis*; BV, control diet containing 1 × 10^7^ CFU/g of *Bacillus velezensis*; and LL, control diet containing 1 × 10^7^ CFU/g of *Lactococcus lactis*.

In general, Proteobacteria, Fusobacteria, and Firmicutes were the three most dominant bacterial phyla in the jejunum of bullfrog (Figure 6A), and their relative abundance was different among experimental groups. The relative abundance of Proteobacteria (the dominant phylum) in NC, BS, BT, and BV groups was 81.84, 61.75, 55.91, and 70.94%, respectively, whereas the dominant phylum in the LL group was Fusobacteria (67.57%). The LL group showed significantly lower abundance of Proteobacteria and higher abundance of Fusobacteria than the NC group (*p* < 0.05; Figure 6B). The highest abundance of Firmicutes was found in the BT group (41.82%) which significantly differed from that of BS, BV, and LL groups (*p* < 0.05). The major genera in the NC group were *Enterobacter* (50.27%), *Plesiomonas* (12.10%), and *Escherichia–Shigella* (6.59%; Figure 7A), and the dominant genera in treated groups were *Enterobacter* (BS, 48.62%), *Bacillus* (BT, 33.86%), *Escherichia–Shigella* (BV, 26.53%), and *Cetobacterium* (LL, 67.20%). The LL group exhibited significantly lower abundance of *Enterobacter* and higher abundance of *Cetobacterium* compared with the NC group (*p* < 0.05; Figure 7B). Moreover, the highest abundance of *Bacillus* and *Escherichia–Shigella* were recorded in the BT and BV groups, respectively.

**FIGURE 8:**
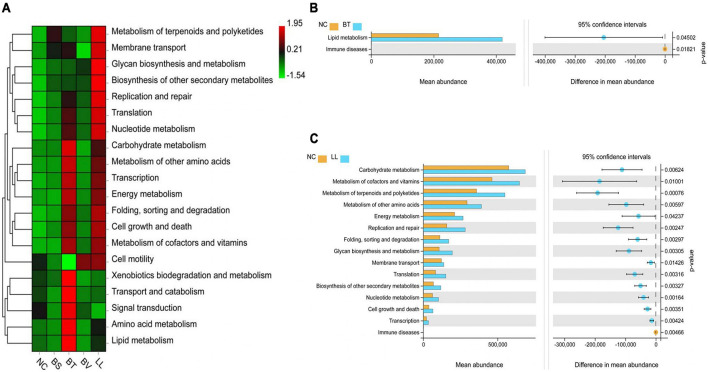
Predicted functional profiles of jejunal microbiota using PICRUSt2 at level 2. **(A)** The heat map analysis among all groups. **(B,C)** Analysis of function difference between NC group and BT (or LL) group. Differences were calculated by Welch’s *t*-test (*p* < 0.05). NC, control diet; BS, control diet containing 1 × 10^7^ CFU/g of *Bacillus siamensis*; BT, control diet containing 1 × 10^7^ CFU/g of *Bacillus tequilensis*; BV, control diet containing 1 × 10^7^ CFU/g of *Bacillus velezensis*; and LL, control diet containing 1 × 10^7^ CFU/g of *Lactococcus lactis*.

Predicted functional analysis of intestinal microbiota by PICRUSt2 revealed the top 20 level-2 KO groups. The KEGG pathways enriched in BS and BV groups were similar to that in the NC group (Figure 8A), whereas BT and LL groups showed a higher abundance of partial KEGG pathways than the NC group. Lipid metabolism was markedly enhanced by the BT diet compared with the NC diet (*p* < 0.05; Figure 8B). Moreover, 14 KEGG pathways including carbohydrate metabolism, metabolism of cofactors and vitamins, metabolism of terpenoids and polyketides, metabolism of other amino acids, energy metabolism, replication and repair, folding, sorting and degradation, glycan biosynthesis and metabolism, membrane transport, translation, biosynthesis of other secondary metabolites, nucleotide metabolism, cell growth and death, and transcription were significantly enriched in the LL group compared with the NC group (*p* < 0.05; Figure 8C).

## Discussion

In the current study, remarkable growth differences were observed among bullfrogs that received SM-based diet for 60 days and reared under the same conditions. Several representative bacterial strains enriched in FG bullfrogs were isolated by standard spread plate method. Presumably, those strains could be adaptive to the gut environment established by a long-term feeding on plant-based diet and might be associated with the growth of bullfrogs. Then, three *Bacillus* species (*B. siamensis*, *B. tequilensis*, and *B. velezensis*) and a lactic acid bacterium (*L. lactis*) were selected as feed additives since the two bacterial categories were among the most common probiotics used in breeding production and experimental research.

In livestock industry and aquaculture, it is challenging to improve the feed utilization of plant-sourced proteins and alleviate their adverse effects on animals. *Bacillus* spp. and lactic acid bacteria are among the most widely used probiotics which exert diverse host-beneficial properties including growth promotion (Yu et al., 2016), immune modulation (Sun et al., 2012), mucosal barrier repair (Lin et al., 2020), disease resistance, etc. (Xia et al., 2019). In the current study, *L. lactis* supplementation improved growth performance, feed efficiency, and nitrogen retention of bullfrogs, and the increase in nitrogen retention was consistent with the trend in whole-body protein content, which agrees with our previous bullfrog study (Lin et al., 2021). This result indicated that the protein synthesis in bullfrogs might be stimulated by *L. lactis* treatment. Similarly, Xia et al. (2018) showed that dietary supplementation of *L. lactis* JCM5805 led to improved weight gain and feed utilization in Nile tilapia (*Oreochromis niloticus*). Also, Li C. et al. (2017) reported enhanced growth of sea cucumber by dietary *L. lactis* LH8 application. However, several researches indicated the lack of growth-promoting effect of *Bacillus* spp. (Reda and Selim, 2015; Yi et al., 2018). In the current study, the three tested *Bacillus* strains (*B. siamensis*, *B. tequilensis*, and *B. velezensis*) had a slight effect on growth of bullfrog, which might be associated with their antibiosis activity, action mechanism, application dosage, and the complex microbiota–host crosstalk (Sahu et al., 2008).

Humoral immunity is a part of innate immune system in aquatic animals, which consisted of complement, LZM, phagocytosis, etc. (Jia et al., 2016). IgM is the key molecule involved in systemic immunity and immune response mechanisms (Liu et al., 2021). C3 and C4 participate in complement pathways, which are crucial to the elimination of pathogens (Alexander and Ingram, 1992). Excessive levels of dietary plant-protein diets increase the risk of immune homeostasis disruption in animals, especially in aquatic animals (Sahlmann et al., 2013). *Bacillus* species and lactic acid bacteria have been proven to stimulate the immune system and maintain the immune homeostasis in animals (Ringø et al., 2018). In this study, *L. lactis* and *B. tequilensis* diets resulted in elevated serum LZM, IgM, and complement levels in bullfrog. Similar results have also been found in olive flounder (*Paralichthys olivaceus*) through dietary supplementation of *L. lactis* I2 (Heo et al., 2013) and in grouper by *L. lactis* application (Sun et al., 2012). Overall, the current study indicated that the immune response of bullfrog might be improved by dietary supplementation of *B. tequilensis* and *L. lactis*.

Intestine morphology is widely used for assessing the intestine development. In the present study, bullfrogs fed the high-SM diet showed a typical SBMIE phenomenon in the jejunum: few and shrunken mucosal folds, as well as narrow muscularis. These degenerative changes might be associated with the inflammatory reaction (Yu et al., 2021). Besides, plant-protein-based diet was proven to induce mucosa damage and infiltration of leukocytes in the lamina propria (Li Y. et al., 2017). In this context, partial separations of tissue were detected in the jejunum of NC, BS, and BV groups. Probiotics have been reported to promote intestinal barrier function through modulation of cytokine production, mucus secretion, macrophage activation, etc. (Anderson et al., 2012). Thus, making use of probiotics conferred a compelling strategy to alleviate adverse impacts caused by plant-protein diet in the gut. For example, Navarrete et al. (2013) reported that dietary *L. lactis* supplementation aided in the restoration of intestinal morphology impaired by SM-based diet in Atlantic salmon (*Salmo salar*). Furthermore, a bullfrog study showed an increase in villus height and thickness by *B. subtilis* application (Lin et al., 2020). Similarly, in the current study, LL and BT diets alleviated the intestinal structural damage caused by high-SM diet. The integrity of intestinal structure is associated with intestinal epithelial permeability (Anderson et al., 2012). Serum DAO activity and D-lactate concentration are two well-established markers for the intestinal mucosal damage (Anderson et al., 2012). In the present study, decreased serum DAO activity indicated the improvement in intestinal integrity and permeability. In addition, intestinal tight junction protein (*zo-1* and *occludin*) were also important components contributing to intestinal physical barrier function (Anderson et al., 2012), and their mRNA expression were up-regulated in bullfrogs fed BT or LL diets as expected. Generally, the results of intestinal morphology, gut permeability, and tight junction proteins collectively indicated that both *B. tequilensis* and *L. lactis* aided in maintaining the intestinal barrier functions and preventing SBMIE.

The intestine epithelium is the main site of nutrient digestion and absorption, the longer and thicker intestinal mucosal folds corresponded to enhanced absorptive surface area (Standen et al., 2016), which could be partially responsible for the increased feed efficiency determined in LL and BT groups. Besides, some probiotics could alter intestinal motility and stimulate the digestive enzyme production, leading to greater digestion efficiency (Anderson et al., 2012), as reported in gibel carp (*Carassius auratus gibelio*) following *B. coagulans* supplementation (Yu et al., 2016), and in Pacific white shrimp after *L. plantarum* application (Zheng et al., 2017). Consistently, in the current study, both jejunal protease and amylase activities of bullfrog were increased by both *B. tequilensis* and *L. lactis* diets, while feed digestibility was increased by *L. lactis* treatment. It has been reported that multiple non-digestible ingredients, including oligosaccharides, disaccharide components, and sugar alcohols, were the main substrates for probiotic growth, and many of these ingredients could be fermented by specific bacteria to short-chain fatty acids, which also conferred health benefits for animals (Sahu et al., 2008). Overall, it could be concluded that intestinal digestive and absorptive functions of bullfrog were improved by both *B. tequilensis* and *L. lactis* supplementation.

The intestinal microbiota is closely tied to the host’s physiological function, nutrient metabolism, and immune homeostasis (Gao et al., 2018; Xia et al., 2018). Diet intake constitutes a pivotal determinant of the compositions of trillions of microorganisms residing in the gut. In the present study, high-SM diet led to the dominance of Proteobacteria, Firmicutes, and Fusobacteria in the jejunum of bullfrog, which was consistent with previous studies on bullfrog (Wang et al., 2020) and freshwater fish species (Desai et al., 2012; Ni et al., 2012). Reportedly, intestinal microbiota disorder was one of the characteristics of enteritis including SBMIE (Merrifield et al., 2010). In the current study, *Enterobacter* or *Escherichia–Shigella* were the major bacterial genera in NC, BS, and BV groups, whereas most bacterial species affiliated to the two genera had been determined as pathogenic bacteria and associated with the inflammatory disorders of animals (Hung et al., 2015; Cao et al., 2017). These results indicated that the long-term SM-based diet might establish favorable conditions for colonization of pathogenic bacteria in bullfrog gut, which might be the main factor that caused intestinal structural damage and microbiota disorder. Probiotics are widely used to modulate the host’s intestinal microbial community; their modes of action include the inhibition of pathogens’ growth by competition for nutrients and adhesion sites, secretion of antibacterial peptides, etc. (Rengpipat et al., 1998; Verschuere et al., 2000). To effectively confer health benefits on the host, it is vitally important for probiotics to adhere and colonize on intestinal mucosa (Alander et al., 1999). In the current study, the BT-fed bullfrogs exhibited an increased abundance of *Bacillus* and decreased abundance of potential pathogens including *Enterobacter*, *Plesiomonas*, and *Escherichia–Shigella*. However, *B. siamensis* and *B. velezensis* diets had a weak effect on the abundance of *Bacillus* in bullfrog jejunum; it suggested that the ability of *Bacillus* species to colonize the intestine varied from strain to strain. Interestingly, the intestinal microbiota in the LL-fed bullfrog was characterized by a bloom of *Cetobacterium*. It was reported that the main components of *L. lactis* were glycerol phosphate, teichoic acid, and some polysaccharides (Vinogradov et al., 2018), which might serve as substrates for *Cetobacterium* growth. However, the concrete mechanism of the symbiotic relationship among microorganisms needs to be further studied. *Cetobacterium* was able to produce vitamin B_12_ and short-chain fatty acids by fermenting carbohydrates and peptides, and thereby promoting the nutrients’ utilization (Finegold et al., 2003; Tsuchiya et al., 2008; Li et al., 2015), as confirmed by the fact that increased nutrient digestibility was obtained in bullfrogs fed *L. lactis* diet.

As an added metabolic “organ” of the host, intestinal microbiota participates in a series of host metabolism steps (Backhed et al., 2004; Zhang et al., 2014). In the current study, analyses of predicted functions of gut microbial communities showed that the pathway of lipid metabolism was stimulated by *B. tequilensis* diet. Zhou et al. (2016) reported that a *Bacillus* strain contributed to decompose cholesterol into coprostanol, and reduced lipid accumulation by stimulating fecal-lipid and bile-acid output. Besides, pathways of metabolisms, including that of carbohydrate, amino acids, energy, cofactors, and vitamins, were significantly enhanced by *L. lactis* diet, and they might be attributed to the effects of *Cetobacterium* on fermenting carbohydrates and peptides as mentioned earlier. Excessive levels of dietary carbohydrate induced metabolic load and stress responses in aquatic animals since most of them were generally considered to be glucose intolerant and poor at utilizing carbohydrates (Qiao et al., 2016; Boonanuntanasarn et al., 2018; Gao et al., 2018). Thus, it is of great significance that gut microbes aid the host in the utilization of carbohydrate and non-digestible ingredients. Collectively, after feeding the SM-based diet supplemented with *L. lactis* for 58 days, the changes in intestinal structure and digestive function, as well as the alterations in gut microbiota, collaborated to promote the gut health, resulting in improved feed utilization and growth performance of bullfrog.

## Conclusion

The beneficial effects of two frog-derived probiotics were determined. Dietary supplementation of *L. lactis* significantly promoted feed utilization and growth performance of bullfrog, and both *L. lactis* and *B. tequilensis* supplementation improved the immune response and alleviated enteritis caused by the high-SM diet. These beneficial effects could be attributed to the improvement in intestinal epithelial integrity and digestive function, as well as the alterations in gut microbiota.

## Data Availability Statement

The datasets presented in this study can be found in online repositories. The names of the repository/repositories and accession number(s) can be found below: GenBank MZ573378, MZ573379, MZ573380, and MZ573381; BioProject PRJNA747862.

## Ethics Statement

The animal study was reviewed and approved by The Committee on the Ethics of Animal Experiments of Jimei University.

## Author Contributions

LW and CZ designed the study. ZW performed the experiment, analyzed data, and wrote the manuscript. KL and KS participated in the experiment design and gave valuable advice. XL and SR contributed to revision of the manuscript. All authors have read and approved the final version of this article.

## Conflict of Interest

The authors declare that the research was conducted in the absence of any commercial or financial relationships that could be construed as a potential conflict of interest.

## Publisher’s Note

All claims expressed in this article are solely those of the authors and do not necessarily represent those of their affiliated organizations, or those of the publisher, the editors and the reviewers. Any product that may be evaluated in this article, or claim that may be made by its manufacturer, is not guaranteed or endorsed by the publisher.
