# Supplementary material for: Effects of Supplementing Intestinal Autochthonous Bacteria in Plant-Based Diets on Growth, Nutrient Digestibility, and Gut Health of Bullfrogs (Lithobates catesbeianus)
Source: Front Microbiol. 2021 Oct 5;12:739572. doi: 10.3389/fmicb.2021.739572 (PMC8524044; doi:10.3389/fmicb.2021.739572)
Supplement: Supplementary file 1 [file Table_1.DOCX]

Supplementary Material

**Table S1.** Formulation and proximate composition of the basal diet (%, dry matter).

| Ingredients | (%) |
| --- | --- |
| Soybean meal | 55.00 |
| Wheat flour | 20.40 |
| Wheat gluten | 5.00 |
| Corn gluten meal | 5.00 |
| Squid paste | 1.50 |
| Fish oil | 3.50 |
| Soybean oil | 3.00 |
| Ca(H_2_PO_4_)_2_ | 2.00 |
| CaCO_3_ | 1.20 |
| Lysine monohydrochloride | 1.58 |
| DL-Methionine | 0.32 |
| Choline chloride | 0.50 |
| L-ascorbate-2-phosphate | 0.10 |
| Mineral premix^a^ | 0.50 |
| Vitamin premix^b^ | 0.20 |
| Y_2_O_3_ | 0.10 |
| Proximate compositions |  |
| Crude protein | 41.10 |
| Crude lipid | 7.90 |
| Total Calcium | 0.86 |
| Total Phosphorus | 0.96 |

^a, b^ Mineral and vitamin premixes were prepared as described by Lin et al. (2020).

**Table S2.** Sequences of primers used for quantitative real-time PCR.

| *Gene* | Forward (5’ → 3’) | Reverse (5’ → 3’) | Annealing temperature (°C) |
| --- | --- | --- | --- |
| *il-17* | TGATAGTCACGCACTGAGTCCG | ATGTTCACCAGCCAGTCAATGC | 60 |
| *tnf-α* | GAGGTGAGAAGAGGCGTGACTT | AGGTCGTCTACACTGGCAAAGA | 60 |
| *il-4* | TAGGCAATCCTGTCTGCGAG | CAGAGTTAGGTGAGGCGGTC | 60 |
| *il-10* | GGAAGGACAGTTCAGCCCAA | CGCTGTGAAACCGAAGTAGC | 60 |
| *occludin* | GATACTCGCTGTCCCAGTCC | ACCAGGAAAAGCCGTGAGAT | 60 |
| *zo-1* | TCTGTACCCACCTCTAGGGC | GCTTGGCTTGGTTTGAGGTG | 60 |
| *β-actin* | CATCCTTCTTGGGTATGGAATCA | TGGCATACAGGTCCTTACGGATA | 60 |

*il*, interleukin; *tnf*, tumor necrosis factor; *zo*, zonula occludens.


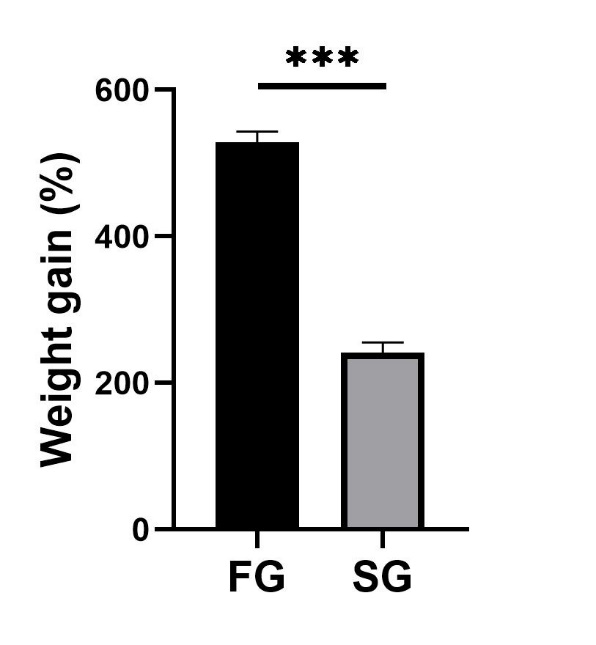


**Figure S1.** The weight gain (%) of fast-growing (FG) and slow-growing (SG) bullfrogs. Data were analyzed by Student’s t-test. Mean values were significantly different: **** P* < 0.001.
